# Assessing the psychological wellbeing and resilience of frontline health workers in Uganda: a cross-sectional study

**DOI:** 10.3389/fpsyt.2025.1568376

**Published:** 2025-09-05

**Authors:** Ahmed M. Sarki, Joseph Atukwatse, Gideon Mbithi, Sabina Odero, Mary Namuguzi, Bernard Mutwiri, Lestine Bitakwitse, Peninah Wachira, Kevinson Mwangi, Eunice Ndirangu-Mugo, Amina Abubakar

**Affiliations:** ^1^ School of Nursing and Midwifery, The Aga Khan University, Kampala, Uganda; ^2^ Family and Youth Health Initiative, Epidemiology and Biostatistics Department, Jigawa State, Nigeria; ^3^ Institute for Human Development, The Aga Khan University, Nairobi, Kenya; ^4^ School of Nursing and Midwifery, The Aga Khan University, Nairobi, Kenya

**Keywords:** healthcare workers, mental health, resilience, psychological wellbeing, Uganda

## Abstract

**Background:**

Health workers in the frontline are the major drivers of health systems in low- and middle-income countries. However, these health workers face both chronic and acute shocks and stressors that expose them to mental health problems which are often overlooked. We examined the prevalence of mental health problems, correlates of psychological functioning, and resilience of frontline health workers in Uganda.

**Methods:**

We conducted a cross-sectional study comprising 1800 frontline health workers across all regions of Uganda from August through October 2023. We used tools such as the PC-PTSD-5, PHQ9, PSS, GAD-7, Oldenburg Burnout Inventory, and CD-RISC-10 to assess PTSD, stress, anxiety, depression, burnout, resilience, and stigma. Correlates of psychological functioning comprising work engagement, quality of life, social support, and attitude were assessed. Data was analyzed using frequency analysis, where applicable, standard logistic regression models were used to examine predictors of common mental disorders among the study participants.

**Results:**

A total of 1800 frontline health workers participated in the study, of whom 59.9% were females. The average age was 38.6 ± 11.4 years. Prevalence of depression, anxiety, and PTSD were 17.5%, 11.1% and 30.3% respectively. Community health workers had higher prevalence of depression (23.7% vs 14.4%, p<0.001), anxiety (13.3% vs 9.9% p=0.029), and PTSD (37.0% vs 26.9%, p<0.001) compared to facility-based workers respectively. Perceived stress, burnout, and negative attitude towards people with mental illness were associated with higher odds of depression. Similarly, these factors had significant association with anxiety and PTSD. Resilience, psychological wellbeing, and perceived social support were associated with lower odds of depression. Also, these factors were protective against anxiety and PTSD, except social support and resilience.

**Conclusion:**

The prevalence of PTSD, depression and anxiety is considerably high among frontline health workers in Uganda. Perceived stress, burnout, and negative attitude towards people with mental illness are associated with higher odds of mental disorders. High scores on resilience, psychological wellbeing, and perceived social support are protective against mental disorders. The mental health and well-being of frontline health workers need to be prioritized by hospital administrators, public health leaders, and policy makers especially in low- and middle-income countries.

## Introduction

Health workers play a crucial role in the functioning of health systems globally. They are relied upon to provide quality, accessible and acceptable health care (1). However, with a projected shortfall of nearly 18 million healthcare workers by 2030, especially in low-resource settings (2), there is a need to enhance health workforce retention. Although many countries are experiencing varying issues in employment, education, and retention of health workforce, the chronic underinvestment in health workers’ well-being and psychosocial support in various countries, contributes to inefficient and overburdened health systems amidst growing population needs (3, 4).

Like other countries, Uganda’s health workforce includes both frontline and non-frontline health workers. The target population for this study was frontline health workers. Operationally, we defined a frontline health worker as a healthcare personnel member who primarily provides direct-patient healthcare care services. These are basically health workers who are primarily in active clinical practice including medical doctors, dentists, nurses, midwives, clinical officers, laboratory technicians/technologists, pharmacists/drug dispensers plus the community-based village health team members (VHTs), as applied to the Ugandan context. For clarity, our study did not include health workers who only practice clinical or community-based direct-patient healthcare service delivery as their secondary work, such as those primarily engaged in healthcare governance and administration, health sciences teaching and training, public health, and health-related research among others. Because of their fulltime engagement in clinical practice and community healthcare service delivery, as opposed to their counterparts in the less demanding non-direct-patient care roles, frontline health workers are arguably the ones most prone to the consequences of underinvestment in health workers’ well-being and psychosocial support.

The World Health Organization has been urging governments to invest in mental health with limited response. The effects of the underinvestment were particularly exposed during the coronavirus disease 2019 (COVID-19) pandemic, which highlighted the fragile healthcare systems in both low-and middle-income countries (LMICs) and high-income settings (HIC) (5–8). The pandemic exposed the insufficient investment in the frontline healthcare workers’ psychosocial support and well-being (9–12). The pandemic’s effects on the health workforce were immense, with extensive adverse implications orchestrated by the existing health inequities. For instance, working at the frontline exposed health workers to various levels of psychological distress, including fear of infection, anxiety, depression, and burnout, among other mental health issues (13–17).

A cross-sectional study conducted among HCWs across three hospitals in Kenya established that 53% of the healthcare workers had experienced depression, 44% anxiety, 41% insomnia, 31% distress, and 45% burnout during the COVID-19 pandemic, with almost half of the participants reporting inadequate training and resources to enhance care (18). Additionally, a study conducted in Vietnam (19) to determine the rates of anxiety, depression, stress and related factors among frontline health workers during COVID-19 reported that 11.5%, 18%, 7.7% of participants had symptoms of anxiety, depression, and stress, respectively with the majority at mild and moderate levels. The risk factors for increased mental health impact among participants as posited by Le Thi Ngoc et al. included experiencing physical symptoms, fear of transmission to family, long working hours, COVID-19 related stigma, and worry when watching media reports about COVID-19 (19).

Psychosocial issues such as burnout have previously been observed to impact the rate of absenteeism and turnover; thus, require immediate intervention. Indeed, nurses who experience high levels of strain or stress are prone to costly mistakes and could likely record higher rates of absenteeism, which impacts health service delivery and quality of care (20). Similarly, Melnyk et al., observed that poor psychosocial well-being among nurses was responsible for poor patient safety and poor health outcomes (21).

In low-resource settings, healthcare personnel work long hours in highly demanding environments, making them susceptible to psychological challenges, including burnout, traumatic experiences, and poor work-life balance. Despite the extensive global awareness of psychosocial issues that health workers face due to stress and burnout, there is a dearth of knowledge regarding psychosocial support and resilience-enhancing strategies for healthcare workers, especially in sub-Saharan Africa (22). Seemingly, although several studies highlight a prevailing dire need for psychosocial support, there is a paucity of evidence and a knowledge lacuna on effective interventions for strengthening resilience and enhancing well-being among frontline healthcare workers in low-resource settings (22). Typically, national governments in such settings are often challenged with fixed budget constraints to meet all the critical needs that exist in their health systems. This leads them to prioritizing only a few that concern direct-patient care delivery (23), and investing in health workers’ psychological wellbeing logically ceases to be top priority. However, there is an urgent need to generate evidence-based psychosocial support guidelines and strategies for promoting healthcare workers’ resilience to ensure sustainable and standard quality of healthcare in resource limited settings too. With limited research focusing on the broad contingent of health workers, this study seeks to redress this gap by engaging nurses, midwives, medical doctors, clinical officers, laboratory technicians, pharmacists/drug dispensers and VHTs, to articulate their experiences on mental health problems, resilience, and psychosocial support needs in Uganda to help underpin interventions informed by their opinions and preferences. The aim of this study was to examine the prevalence of mental health problems, correlates of psychological functioning and resilience of frontline health workers in Uganda. The implication is to inform policy change to promote, generally, mental health wellbeing of frontline health workers in Uganda and other resource limited countries.

## Methods

### Study design

This was a cross-sectional survey of frontline health workers, both facility-based and community-based, in Uganda conducted from August to October 2023. Following our definition of frontline health workers, the study population was divided into three main categories: 1) nurses and midwives, 2) other facility based frontline health workers, i.e., doctors, clinical officers, laboratory technicians/techonologists, pharmacists/pharmaceutical technicians/drug dispensers and dentists, and 3) community-based health workers, the village health teams (VHTs). Eligible participants were health workers who had worked for more than 6 months in their respective category at the time of data collection and had to be aged at least 18 years. Health workers who did not speak either English or any of the six main local languages in the selected district (i.e., Luganda, Lusoga, Dhopadhola, Runyankole/Rukiga, Lukonzo, Acholi, or Lugbarati) were excluded. Students and interns were also excluded.

### Sample size calculation

We used STATA/SE 14.1 to compute the sample size (StataCorp, 2015) using the formula:


$$\text{n}={(Z_{1-\frac{\text{α}}{2}})}^{2}p\left(1-p\right)/d^{2}$$

where: n is the sample size,

Z is the statistic for the level of confidence,

p is the prevalence,

and d is the margin error.

We estimated the prevalence of mental health workers to be 22.9% based on our earlier work in Kenya (23). We used a 95% confidence level (z = 1.96), a 2% margin of error and obtained a sample size of 1,696. After adjusting for a 10% non-response rate, a sample of 1,800 participants was projected as sufficient. We distributed this sample size in equal proportions for the three broad categories to cater for the apparent picture that nurses and midwives form the biggest portion of all health professionals in Uganda and elsewhere in the world.

### Sampling and data collection procedures

We used multi-stage stratified random sampling to recruit the study participants. First, we used proportionate to size sampling to allocate the sample, by cadre, to each region. We allocated the sample size of each category of frontline health workers to each region based on the number of health care workers in the specified region to avoid over/under sampling by region (24).

#### Recruitment of facility-based participants

Based on the 2017 National Health Facility Master List for Uganda, there were 6,404 registered health facilities (both public and private) in the country (25). The central region had the highest proportion of the facilities at 45.5% (n=2,914), followed by the western region with 1,367 health facilities (21.3%), then the eastern region with 1,196 facilities (18.7%) and lastly the northern region with 927 registered health facilities (14.5%) (25). Assuming the number of health facilities in each geographical area directly relates to the number of health professionals in the same area, we used the same percentages to distribute the study sample.

This sample allocation provided the number of frontline health professionals, by cadre, that was required from each region for the study. Further, we purposively selected two districts from each region targeting districts with the highest number of registered health facilities as per the national health facility master list of 2017 to recruit the study participants (25). The selected districts were Kampala and Wakiso (Central region), Kasese and Kabale (Western region), Tororo and Jinja (Eastern region), Arua and Gulu (Northern region).

Ten health facilities were purposefully selected from each district: the major government owned health facility (e.g., district hospital, regional referral hospital, or National referral hospital), then randomly selected nine other registered health facilities. Of the nine, six were government owned facilities and the other three were private for all districts except for Kampala and Wakiso. For the latter two districts, we selected 6 private and 3 government owned facilities out of the 9 other facilities, given that these two districts largely had more private owned facilities than government owned (25).

A team of 10 well trained experienced enumerators together under support supervision of a field supervisor and a project coordinator would then approach the health workers found on duty on the day and time of visit to each facility, following prior notification to the administration, and seek their consent for their participation in the study. Whereas this conforms to convenience sampling at individual participant level, we still had a significant level of random sampling since the data collection team did not have influence on who to find at the facility by the time of visit. To enable representativeness of the sample across the facilities, peripheral (lower level) health facilities were visited first, then major (higher level) facilities later. This was to maximize chance for participants from lower-level facilities before visiting the major facilities for each district by participant category given fewer healthcare workers in lower-level facilities. Where a selected facility opted out, was not able to participate for any reason or was no longer in existence by the time of data collection, a matching facility by type of ownership and level of operation in the same district was selected at random for replacement.

#### Recruitment of VHTs

The VHT participants were proportionately distributed across all the four regions 27.9% (Central region), 26.0% (Eastern region), 25.4% (Western region), and 20.7% (Northern region). Given that the number of VHTs in each geographical region of Uganda is determined by the population size of that area, the probability proportionate sampling of VHTs was guided by the regional population distribution estimates (26).

Like with the facility-based frontline health workers, we mobilized VHTs and met them at a sub-county or division level with help of their coordinators in the area. For representativeness, we equally distributed the sample size allocated to each region across the two districts and the number of sub-counties or divisions in each district. During participant mobilization we ensured that at least each sub-county or division was represented in the sample while considering voluntariness in participation. The mobilized participants would then be met at an agreed upon central place, such as the sub-county/division headquarters or health facility, on a prior communicated date and time for consent and data collection.

**Figure 1:**
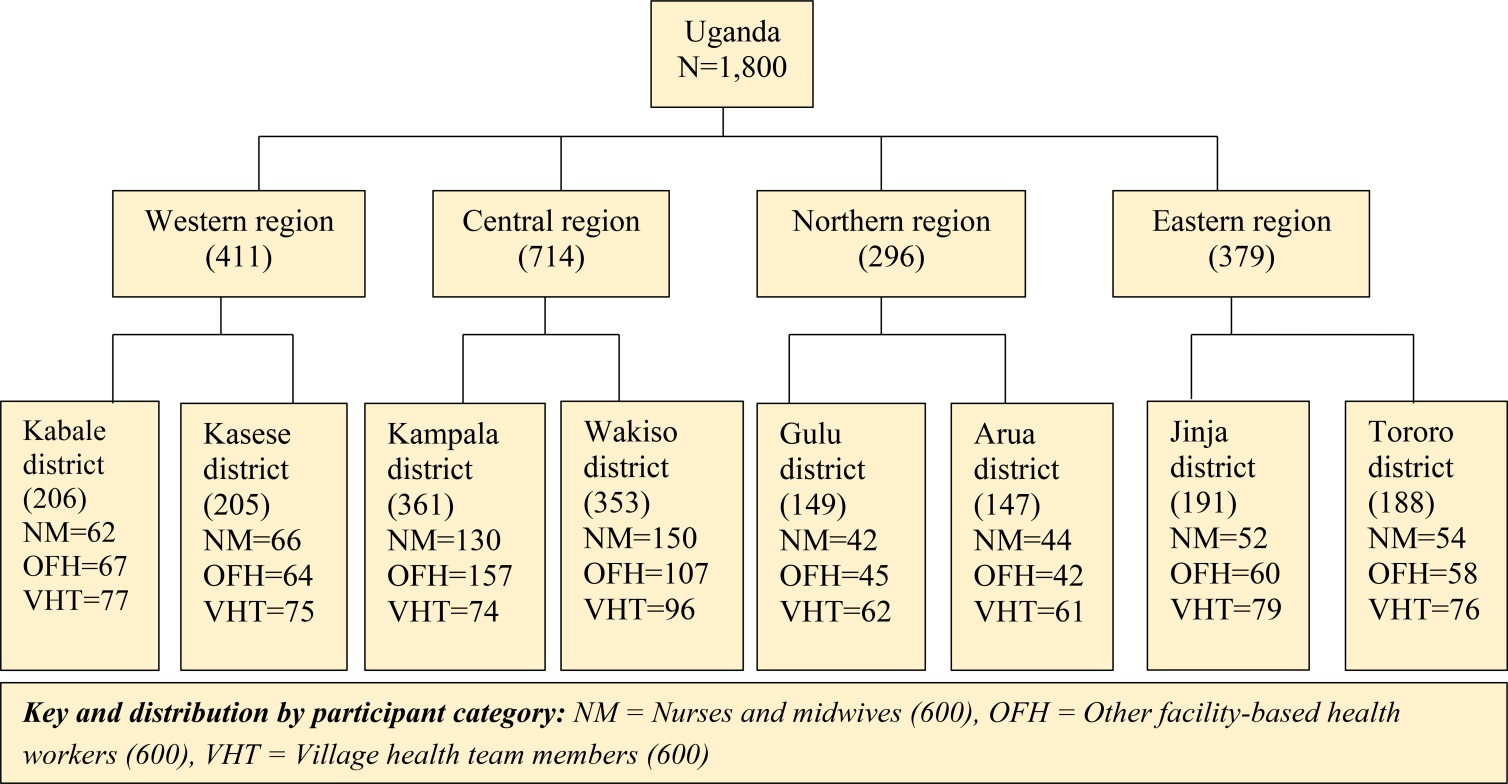
Sample size distribution up to district level for all three participant categories.

Ultimately, we enrolled 1,800 study participants across the four geographical regions and eight selected districts countrywide, and across the three broad participant categories as shown in **Figure 1**.

### Tools and outcome measures

The tools highlighted in **Table 1** above have been pretested on 60 participants in five health centers and one subcounty in Mukono District, Central Uganda. The health facilities included one district hospital [government owned], three health center III’s [one government, one private-not-for-profit, one private-for-profit], and one health center II [government owned]. Despite these being validated tools, the purpose of pretesting them was to ensure the different items would be contextually understood by the intended participants, especially upon translation in the local languages, and to ensure feasibility of the data collection procedures as had been proposed by improving where necessary.

**Table 1:** Tools, descriptions, outcome measures, and, and psychometric properties.

Tool	Brief description of the tool	Primary outcomes	Cronbach’s alpha (95% CI)
Patient Health Questionnaire-9 (PHQ-9)	Assesses severity of depression symptoms based on 9 questions	Depression	0.76 (0.73 to 0.77)
Generalized Anxiety Disorder-7 (GAD-7)	Assesses severity of generalized anxiety disorder symptoms based on 7 questions	Anxiety	0.80 (0.78 to 0.82)
Primary Care PTSD Screen for DSM-5 (PC-PTSD-5)	Screens for PTSD symptoms based on DSM-5 criteria	Posttraumatic Stress Disorder	0.76 (0.74 to 0.78)
Perceived Stress Scale (PSS)	Measures perception of stress in one’s life	Perceived Stress	0.62 (0.60 to 0.65)
Oldenburg Burnout Inventory	Measures burnout in the workplace	Burnout	0.69 (0.67 to 0.71)
Connor-Davidson Resilience Scale 10 (CD-RISC-10)	Assesses resilience in bouncing back from stress	Resilience	0.80 (0.79 to 0.82)
Utrecht Work Engagement Scale (UWES)	Measures engagement in one’s work	Work Engagement	0.71 (0.69 to 0.73)
WHO-5 Item Wellbeing Index	This is a 5-item tool that assesses psychological well-being	Psychological Wellbeing	0.80 (0.78 to 0.81)
Opening Minds Scale for Health Care Providers	Measures stigma related to mental health among healthcare providers	Stigma	0.73 (0.71 to 0.74)
Multidimensional Scale of Perceived Social Support (MSPSS)	Measures perceived social support from various sources	Social Support	0.84 (0.83 to 0.86)

### Statistical analysis

Summary statistics were presented as mean (SD) and frequency (%) for continuous and categorical variables, respectively. We compared the means of continuous variables between the facility versus the community-based health workers using the two-sample t-test method. The distributions of categorical variables were compared using Pearson’s chi-squared test or Fisher’s exact test as appropriate.

We investigated the factors associated with depression, anxiety, and PTSD using three separate logistic regression models. We used a *P-*value ≤ 0.25 in a backwards stepwise variable selection procedure to identify the variables to be included in the final multivariable models. For all statistical analyses, we used R statistical software (version 4.3.2) and a significance level of 5%.

### Ethical considerations

This study received ethical approval from The AIDS Support Organization’s Research Ethics Committee in Uganda under reference number: TASO-2023-238. Administrative clearance was obtained from the respective district administrative authorities led by the district health officers and health facility heads.

Informed consent was obtained from all participants prior to implementation of data collection procedures in the field, all participants were given a small compensation for their time in the study as guided by the Uganda National Council for Science and Technology.

## Results

### Summary of study participants

There were a total of 1800 participants who were engaged in the study. **Table 2** shows the characteristics of the study participants. Furthermore, statistical group comparisons between facility-based workers and village health teams-VHTs (community health volunteers) are shown in **Table 2**. The overall mean age was 38.6 years (*SD = 11.4*), most of the participants were female (59.9%), married (55.5%), Christians (92.2%), and facility-based (66.7%).

**Table 2:** Sociodemographic characteristics of the study participants stratified by health worker category.

Characteristic	Overall, N = 1,800*^1^*	Community health workers, N = 600*^1^*	Facility-based workers, N = 1,200*^1^*	P-value*^2^*
Age (years)	38.62 (11.36)	44.57 (11.56)	35.64 (10.02)	<0.001
Gender				0.061
* Male*	722 (40.1%)	259 (43.2%)	463 (38.6%)	
* Female*	1078 (59.9%)	341 (56.8%)	737 (61.4%)	
Highest education level				<0.001
* Primary*	104 (5.8%)	104 (17.3%)	0	
* Secondary*	377 (20.9%)	362 (60.3%)	15 (1.3%)	
* Certificate*	481 (26.7%)	67 (11.2%)	414 (34.5%)	
* Diploma*	569 (31.6%)	47 (7.8%)	522 (43.5%)	
* Bachelor or above*	269 (14.9%)	20 (3.3%)	249 (20.8%)	
Marital status				<0.001
* Single*	665 (36.9%)	145 (24.2%)	520 (43.3%)	
* Married*	999 (55.5%)	362 (60.3%)	637 (53.1%)	
* Divorced/widowed/widower*	136 (7.6%)	93 (15.5%)	43 (3.6%)	
Religion				0.069
* Christians*	1651 (92.2%)	540 (90.6%)	1111 (93.0%)	
* Muslim*	139 (7.8%)	56 (9.4%)	83 (7.0%)	
Cadre				–
* Medical doctor*	125 (7.2%)	–	125 (11.1%)	
* Nurse/midwives*	603 (34.9%)	–	603 (53.4%)	
* Clinical officer*	178 (10.3%)	–	178 (15.8%)	
* Village health teams*	600 (34.7%)	600 (100.0%)	–	
* Laboratory technician*	223 (12.9%)	–	223 (19.8%)	
Work duration (years)	10.37 (12.50)	11.02 (6.92)	10.05 (14.50)	0.121
Working hours/day				<0.001
* 0-11*	1613 (89.6%)	593 (98.8%)	1020 (85.0%)	
* >12*	187 (10.4%)	7 (1.2%)	180 (15.0%)	
Do you have a side work (yes)	508 (28.4%)	282 (47.6%)	226 (18.9%)	<0.001
Health facility management				<0.001
* Public*	1394 (77.9%)	549 (92.9%)	845 (70.5%)	
* Private*	396 (22.1%)	42 (7.1%)	354 (29.5%)	
Health Insurance (yes)	190 (10.6%)	1 (0.2%)	189 (15.8%)	<0.001

*^1^* Mean (SD); n (%).

*^2^* Two Sample t-test; Pearson’s Chi-squared test.

The VHTs had a relatively higher (P<0.001) mean age (M = 44.6, *SD = 11.6*) than the facility-based workers (M = 35.6, *SD = 10.0*). There was no significant difference in terms of the mean work duration between the two comparison groups [P = 0.121]. Additionally, there was no significant difference between the number of participants in the two groups, when you compare them by religion [P = 0.069]. Furthermore, the results show a significant difference in level of education, marital status, working hours, having side work, and the type of health facility (**Table 2**).

### Prevalence of common mental health problems

**Table 3** summarizes the prevalence estimates for depression, post-traumatic stress disorder, and anxiety symptoms. In terms of severity, community health workers had higher prevalence of depression (23.7% vs 14.4%, p<0.001), anxiety (13.3% vs 9.9% p=0.029), and PTSD (37.0% vs 26.9%, p<0.001) compared to facility-based workers respectively.

**Table 3:** Prevalence of depression, anxiety, and PTSD among the study participants.

Characteristic	Overall N = 1,800 (95 CI)*^1,2^*	Community health workers N = 600 (95 CI)*^1,2^*	Facility-based workers N = 1,200 (95 CI)*^1,2^*	*P*-value*^3^*
Depression (PHQ-9 ≥ 10)	17.5 (15.8, 19.4)	23.7 (20.4, 27.3)	14.4 (12.5, 16.6)	<0.001
Severity of depression				<0.001
* None (0-4)*	50.6 (48.2, 52.9)	42.8 (38.8, 46.9)	54.4 (51.5, 57.3)	
* Mild (5-9)*	31.9 (29.8, 34.2)	33.5 (29.8, 37.5)	31.2 (28.6, 33.9)	
* Moderate (10-14)*	13.8 (12.3, 15.5)	18.8 (15.8, 22.2)	11.3 (9.6, 13.3)	
* moderately severe (15-19)*	3.3 (2.5, 4.2)	4.5 (3.0, 6.6)	2.7 (1.9, 3.8)	
* Severe (20-27)*	0.4 (0.2, 0.8)	0.3 (0.1, 1.3)	0.4 (0.2, 1.0)	
Anxiety (GAD-7 ≥ 10)	11.1 (9.7, 12.6)	13.3 (10.8, 16.4)	9.9 (8.3, 11.8)	0.029
Severity of anxiety				0.001
* None (0-4)*	61.2 (58.9, 63.4)	54.8 (50.7, 58.9)	64.3(61.5, 67.0)	
* Mild (5-9)*	27.8 (25.7, 29.9)	31.8 (28.2, 35.8)	25.8 (23.3, 28.3)	
* Moderate (10-14)*	9.3 (8.0, 10.7)	11.2 (8.8, 14.0)	8.3 (6.9, 10.1)	
* Severe (15-21)*	1.8 (1.2, 2.5)	2.2 (1.2, 3.8)	1.6 (1.0, 2.5)	
PTSD	30.3 (28.2, 32.5)	37.0 (33.1, 41.0)	26.9 (24.4, 29.5)	<0.001
Positive screen for comorbid depression, anxiety, and PTSD	4.3 (3.4, 5.3)	5.5 (3.9, 7.7)	3.7 (2.7, 4.9)	0.070

*^1^* Prevalence (%).

*^2^* CI = Confidence Interval.

*^3^* Pearson’s Chi-squared test; Fisher’s exact test.

The overall prevalence of positive screen for comorbidity for depression, anxiety, and PTSD symptoms was 4.3%, There was no statistically significant difference in the prevalence of comorbid depression, anxiety, and PTSD between the community and facility-based workers (P=0.070.).

### Other psychological outcomes

**Table 4** provides a summary of other psychological outcomes that were measured in the study. Overall mean of the scores is provided, as well as mean comparison of scores between VHTs, and facility-based worker. Facility based workers had higher significant burnout scores (M = 37.6, SD = 4.6), compared to community-based workers (M = 36.5, SD = 4.4), *p <0.001*. In addition, there was a significant difference in terms of resilience scores between the two groups, with facility-based workers having higher resilience scores (M = 27.8, SD = 5.8), compared to VHTs (M = 25.9, SD = 6.3), *p <0.001*.

**Table 4:** Comparison of mean scores for the other psychological measures between community and facility-based health workers.

Characteristic	Overall, N = 1,800*^1^*	Community health workers, N = 600*^1^*	Facility-based workers, N = 1,200*^1^*	P-value*^2^*
Perceived Stress Scale score	16.69 (5.46)	17.02 (5.14)	16.53 (5.61)	0.086
Oldenburg Burnout Inventory score	37.22 (4.38)	36.47 (3.81)	37.59 (4.60)	<0.001
Connor-Davidson Resilience Scale 10	27.13 (6.04)	25.85 (6.29)	27.77 (5.80)	<0.001
Utrecht Work Engagement score	44.13 (7.80)	44.31 (7.82)	44.04 (7.79)	0.330
WHO-5 Item Wellbeing Index score	15.11 (5.85)	14.83 (5.98)	15.25 (5.78)	0.130
Opening Minds Scale for Health Care Providers	62.04 (6.35)	63.84 (6.73)	61.15 (5.95)	<0.001
Multidimensional Scale of Perceived Social Support	67.81 (7.99)	67.90 (7.84)	67.76 (8.07)	0.639

*^1^* Mean (SD)

*^2^* Two-sample t-test.

Furthermore, VHTs had relatively high significant scores in terms of having negative attitude towards people with mental illness (M = 63.8, *p <0.001*). There was no significant difference between the two groups in terms of perceived stress, work engagement, psychological wellbeing, quality of life, and perceived social support (**Table 4**).

### Regression analysis

**Table 5** shows the results of the multivariable regression model showing both protective and risk factors associated with depression, anxiety, and post-traumatic stress disorder.

**Table 5:** Multivariable logistic regression models result of factors associated with depression, anxiety, and PTSD.

Characteristic	Depression			Anxiety			PTSD		
	OR*^1^*	95% CI*^1^*	*P*-value	OR*^1^*	95% CI*^1^*	*P*-value	OR*^1^*	95% CI*^1^*	*P*-value
Highest education level									
*Primary*	*Ref.*	*Ref.*		*Ref.*	*Ref.*		—	—	—
*Secondary*	1.40	0.77, 2.63	0.277	2.61	1.18, 6.51	0.026	—	—	—
*Certificate*	2.14	1.55, 4.46	0.039	1.52	0.56, 4.36	0.419	—	—	—
*Diploma*	1.71	0.82, 3.65	0.158	1.75	0.64, 5.05	0.285	—	—	—
*Bachelor or above*	1.23	0.53, 2.85	0.626	1.87	0.63, 5.80	0.262	—	—	—
Cadre									
*Medical doctor*	*Ref.*	*Ref.*		*Ref.*	*Ref.*		*Ref.*	*Ref.*	
*Nurse/midwives*	0.67	0.33, 1.41	0.284	1.63	0.69, 4.17	0.283	0.73	0.47, 1.15	0.171
*Clinical officer*	0.97	0.45, 2.12	0.930	0.90	0.33, 2.51	0.835	0.76	0.44, 1.31	0.319
*Village health teams*	1.72	0.79, 3.82	0.177	1.60	0.59, 4.54	0.361	1.28	0.82, 2.01	0.285
*Laboratory technician*	0.60	0.28, 1.32	0.199	1.24	0.48, 3.34	0.660	1.08	0.66, 1.80	0.759
Work experience (years)	0.98	0.96, 1.00	0.053	—	—	—	—	—	—
Psychological measures									
Perceived Stress	1.13	1.10, 1.17	<0.001	1.15	1.11, 1.20	<0.001	1.10	1.08, 1.13	<0.001
Burnout	1.08	1.04, 1.12	<0.001	1.10	1.06, 1.15	<0.001	1.02	0.99, 1.05	0.176
Resilience	0.96	0.94, 0.99	0.004	0.97	0.94, 1.00	0.022	—	—	—
Psychological wellbeing	0.94	0.91, 0.96	<0.001	0.91	0.88, 0.94	<0.001	0.98	0.96, 1.00	0.016
Stigma	1.04	1.02, 1.07	<0.001	1.04	1.02, 1.07	0.001	1.02	1.00, 1.04	0.048
Perceived social support	0.98	0.97, 1.00	0.037	—	—	—	0.99	0.97, 1.00	0.041

*^1^* OR, Odds Ratio; CI, Confidence Interval. Variable selection was based on a backward stepwise selection procedure where variables with P-value ≤ 0.25 were included in the final multivariable models. — represent variables that were not included in the multivariable logistic regression analyses for the respective outcome.

Variables associated with high odds of depression include having certificate education (OR 2.14, 95% CI 1.55, 4.46), reporting high levels of perceived stress (OR 1.13, 95% CI 1.10, 1.17), stigma (OR 1.04, 95% CI 1.02, 1.07) and burnout (OR 1.08, 95% CI 1.04, 1.12) (**Table 5**). Resilience (OR 0.96, 95% CI 0.94, 0.99), perceived social support (OR 0.98, 95% CI 0.97, 1.00) and psychological wellbeing (OR 0.94, 95% CI 0.91, 0.96) were associated with lower odds of depression (**Table 5**).

Furthermore, variables associated with increased odds of anxiety included having a secondary level of education (OR 2.61, 95% CI 1.18, 6.51) relative to primary education, reporting high levels of perceived stress (OR 1.15, 95% CI 1.11, 1.20), stigma (OR 1.04, 95% CI 1.02, 1.07) and burnout (OR 1.10, 95% CI 1.06, 1.15). Additionally, high psychological wellbeing is associated with lower odds of anxiety (OR 0.91, 95% CI 0.88, 0.94).

High perceived stress scores (OR 1.10, 95% CI 1.08, 1.13) and stigma (OR 1.02, 95% CI 1.00, 1.04) were associated with high odds of PTSD. Protective factors associated with low odds of PTSD include high psychological wellbeing (OR 0.98, 95% CI 0.96, 1.00), and perceived social support (OR 0.99, 95% CI 0.97, 1.00) (**Table 5**).

## Discussion

This study provides important insights about the burden and correlates of mental health problems among health care workers in Uganda. A total of 1800 participants were engaged in the study, in which 1200 were facility-based workers, and 600 were community-based workers. The overall prevalence of depression, anxiety, and post-traumatic stress disorder was 17.5%, 11.1%, and 30.3% respectively. Comparing these findings with other studies in similar setting is limited since there are few studies targeting mental health among frontline health workers in the region (27), variations in methodology in the few studies, and differences in measurement outcomes and tools used (28). A global systematic review of prevalence of depression and anxiety among health care workers reported a pooled prevalence of 22.8%, and 23.2% respectively (29). However, the review assessed studies done during the COVID-19 pandemic, while our study data collection was done post-COVID.

Community-based workers experienced significantly worse outcomes across all primary measures of the study (depression, anxiety, and PTSD) compared to their facility-based counterparts (see **Table 3**). Prior assessments have shown that community health volunteers in Uganda face multiple challenges (30–32). The unique challenges faced by community health volunteers in Uganda, include limited resources, lack of or low renumeration, lack of uniforms, inadequate skills, exposure to trauma, social isolation, stigma, and high workload, may collectively contribute to their increased risk of experiencing depression, anxiety, and PTSD compared to health care facility-based workers (31). The myriads of challenges faced by community health volunteers are not just unique to Uganda but have been reported in other developing countries including Zimbabwe (33) Kenya (34, 35) among other sub-Saharan countries (34). Ogutu et al. has suggested the following measures to be put in place to safeguard the wellbeing and performance of community health volunteers; adequate and timely remuneration for CHWs, improved and adequate security measures at the community level, appropriate holistic training, adequate supportive supervision, and ensuring a satisfactory supply of resources and supplies (36).

While facility-based healthcare workers exhibit relatively lower levels of mental health issues compared to community-based volunteers in the current study, this does not suggest that their mental health outcomes are satisfactory. The challenges they face including the demanding nature of their work, combined with factors like high expectations, limited time, capacity constraints, and insufficient social support affect their mental well-being (37). Additionally, in the current study, we identified burnout, and participants’ negative attitude towards people with mental health illnesses as key risk factors predicting mental health problems among health workforces. The consequences of these factors among healthcare workers extend beyond their individual well-being. It can negatively impact patient care, as health care workers may find it challenging to maintain the level of attentiveness, empathy, and focus required in their roles. Therefore, it is crucial to provide multifaceted support programmes including encouraging work life balance (38), providing mental health support, promoting physical activity, fostering supportive work environment, addressing workplace stressors, promoting continuing education, providing flexible work schedules, and encouraging self-care to promote positive wellness among healthcare workers (39, 40).

In the current study, resilience, good psychological well-being, a high quality of life, and perceived social support emerged as crucial protective factors safeguarding healthcare workers from experiencing adverse mental health outcomes. These findings are crucial and consistent with previous research (41, 42). Good psychological well-being is not just an indicator of positive mental health but is reflected in positive emotion and behaviors as well. Social support and good psychological well-being are crucial as it ensures one forms positive relationships, manage social pressures independently, have a sense of purpose and meaning in life, and continuously develop their existing abilities to achieve personal growth (43). Social support and good psychological wellbeing have also been pointed out to foster resilience among health care workers. A systematic review identified key factors influencing resilience in health professionals: individual factors like having a higher purpose and being self-determined, environmental and organizational factors such as workplace culture, personal approaches to professional challenges like self-reflection, and effective educational interventions like resilience workshops (44). Hence, creating a supportive and conducive work environment is important as it fosters resilience and psychological well-being among healthcare workers as well as enhancing patient care quality.

Furthermore, although our findings suggest that frontline health workers in Uganda have considerably high levels of burnout (45), facility-based workers stand a significantly higher risk than their community-based counterparts. This could be hinged on relatively heavier mandatory workload facility-based workers are faced with on a full-time basis compared to VHTs who largely play their role in healthcare delivery on a part-time basis. This partly concurs with work by Abhiram et al. which suggests a positive relationship with separation from one’s family and OLBI scores (46), and partly with Aguwa, Nduka, and Arinze-Onyia’s findings that higher levels of education increase risk for burnout (47). This calls for instituting reasonably shorter working hours a day, in contrast to the current state of working hours for facility-based workers as seen in **Table 2**, by human resource managers and urging personnel to take their annual leave breaks when due.

Respondents generally demonstrated high resilience based on the cut-off of 25.5 (48). Higher scores among facility-based workers compared to VHTs seem to be synonymous with the findings of Sánchez-Zaballos and Mosteiro-Díaz (49). The latter found that doing shifts which do not involve night duty is associated with lower resilience scores. Typically, this is applicable to VHTs, who work during the day and for shorter hours (**Table 2**), although, we must consider that they are not salaried workers. However, there is need for targeted policy-based interventions to enhance resilience among VHTs.

Given the stigma surrounding mental illness, it is unsurprising that in this study, VHTs acknowledge negative attitudes towards people with mental illnesses. However, the discourse of stigma and mental health is not unique to Uganda or sub-Saharan Africa, for example, de Filipis, El Hayek, & Shalbafan posit that stigma associated with people suffering from mental health illnesses including their providers and caregivers is a global concern with far-reaching consequences (50). The Bloom’s taxonomy of learning addresses the affective domain, concerned with shaping professional attitude (51, 52), as applied to health professionals during their training, and these formed the facility-based category of health workers in this study. Agreeably, being a health worker compared to being lay and having additional training in mental health, both of which closely relate to facility-based workers, have been highlighted among other factors associated with better attitudes towards persons with mental illness (53). VHTs, on the other hand, are not primarily trained as health professionals. They are lay individuals who are democratically chosen by their own communities to promote health and wellbeing of all village members as per the Uganda’s healthcare system (32). The findings call for additional efforts in creating awareness through regular training focused on mental health for VHTs.

### Limitations

Some of the limitations of the study include the cross-sectional nature of the study where causality cannot be inferred. Further, these findings may not represent responses from frontline health workers deployed in extremely busy and more restricted clinical units and facilities to which the data collection team could not readily access participants for interviews. These included emergency departments, intensive care units, operating theatres, and some private hospitals with high patient volumes against low staff numbers. Due to their busier nature of work, such health workers could have higher indices of burnout and other forms of psychological discomfort that could render our statistical estimates lower than actual. However, the health workers that participated in the current study are representative of the heath workers in the study settings.

## Conclusion

In conclusion, prevalence of PTSD, depression and anxiety is considerably high among frontline health workers in Uganda. Lower qualifications, perceived stress, burnout, and negative attitude towards people with mental illness are associated with higher odds of mental disorders. High scores on resilience, psychological wellbeing, and perceived social support are inversely associated with mental disorders. Consequently, mental health and psychological well-being of frontline health workers need to be prioritized by hospital administrators, public health leaders, and policy makers especially in in low- and middle-income countries. These findings provide opportunities for targeted interventions to improve psychological wellbeing of frontline health workers, which in turn could improve patient outcomes.

## Data Availability

The raw data supporting the conclusions of this article will be made available by the authors, in due consideration of the university’s data sharing policies, without undue reservation. Requests to access the datasets should be directed to the Research Office, Aga Khan University Kenya through research.supportea@aku.edu OR akukenya.researchoffice@aku.edu.
